# Safer disclosure of HIV serostatus for women living with HIV who experience or fear violence: a systematic review

**DOI:** 10.7448/IAS.18.6.20292

**Published:** 2015-12-01

**Authors:** Caitlin E Kennedy, Sabina Haberlen, Avni Amin, Rachel Baggaley, Manjulaa Narasimhan

**Affiliations:** 1 Department of International Health, Johns Hopkins Bloomberg School of Public Health, Baltimore, MD, USA; 2 Department of Epidemiology, Johns Hopkins Bloomberg School of Public Health, Baltimore, MD, USA; 3 Department of Reproductive Health and Research, World Health Organization, Geneva, Switzerland; 4 Department of HIV/AIDS, World Health Organization, Geneva, Switzerland

**Keywords:** disclosure, violence, gender-based violence, review, systematic

## Abstract

**Introduction:**

Supporting individuals as they disclose their HIV serostatus may lead to a variety of individual and public health benefits. However, many women living with HIV are hesitant to disclose their HIV status due to fear of negative outcomes such as violence, abandonment, relationship dissolution and stigma.

**Methods:**

We conducted a systematic review of studies evaluating interventions to facilitate safer disclosure of HIV status for women living with HIV who experience or fear violence. Articles, conference abstracts and programme reports were included if they reported post-intervention evaluation results and were published before 1 April 2015. Searching was conducted through electronic databases for peer-reviewed articles and conference abstracts, reviewing websites of relevant organizations for grey literature, hand searching reference lists of included studies and contacting experts. Systematic methods were used for screening and data abstraction, which was conducted in duplicate. Study quality (rigor) was assessed with the Cochrane risk of bias tool.

**Results:**

Two interventions met the inclusion criteria: the Safe Homes and Respect for Everyone cluster-randomized trial of combination HIV and intimate partner violence (IPV) services in Rakai, Uganda, and the South Africa HIV/AIDS Antenatal Post-Test Support study individual randomized trial of an enhanced counselling intervention for pregnant women undergoing HIV testing and counselling. Both programmes integrated screening for IPV into HIV testing services and trained counsellors to facilitate discussions about disclosure based on a woman's risk of violence. However, both were implemented as part of multiple-component interventions, making it impossible to isolate the impact of the safer disclosure components.

**Conclusions:**

The existing evidence base for interventions to facilitate safe HIV serostatus disclosure for women who experience or fear violence is limited. Development and implementation of new approaches and rigorous evaluation of safe disclosure outcomes is needed to guide programme planners and policy makers.

## Introduction

HIV serostatus disclosure has been associated with many potential benefits. People who disclose their HIV status may receive social support and experience reduced stigma [1], which may in turn lead to other positive outcomes for them, their partners and their families, such as engagement in HIV prevention services (including prevention of mother-to-child transmission [PMTCT]) and uptake of and adherence to HIV care and treatment services [2]. Disclosure to sexual partners could also increase rates of HIV testing and reduce transmission risk behaviours [3], including increasing the use of antiretroviral treatment [4] or pre-exposure prophylaxis (PrEP) [5] to prevent HIV transmission in sero-discordant couples.

Despite these potential benefits, evidence from a number of studies and several reviews shows that a substantial number of women are hesitant to disclose their HIV status due to fear of negative outcomes such as violence, abandonment, relationship dissolution, stigma, loss of children or loss of their home [6,7]. Violence is common in the lives of women. Globally, 35% of women age 15 and older are estimated to have experienced physical or sexual violence by an intimate partner or non-partner sexual violence in their lifetime [8]. A recent survey of 832 women living with HIV from 94 countries found that participants identified violence as a significant concern related to their sexual and reproductive health [9]. Violence can be both a risk factor for and an outcome of HIV infection [8]. Women who are currently in violent relationships may be at risk of further violence that is triggered by HIV serostatus disclosure, while other women who have not previously experienced violence may newly experience it as an outcome of disclosure. A 2003 systematic review found that following HIV serostatus disclosure to a partner between 3.5 and 14.6% of women across studies reported experiencing negative reactions, including violence [6]; another non-systematic review also found that violence may occur around disclosure [10]. More recent studies from Ethiopia, Nigeria and Zimbabwe have found that large proportions of women report negative reactions, including violence, as outcomes of disclosure and that women who disclosed were at increased risk of physical and emotional violence compared to those who had not [11–13]. Further, a systematic review of studies from sub-Saharan Africa found that a history of domestic violence is associated with non-disclosure of HIV serostatus among pregnant and postpartum women [14]. Uptake of HIV testing services among women has increased, especially among pregnant women in the context of PMTCT programmes. However, fear of disclosure and experience of violence and other traumatic or stressful life events after disclosure of HIV status have been associated with lower adherence to antiretroviral treatment for women's own health and for PMTCT [15–19]. Fear and experience of violence related to HIV serostatus disclosure may also prevent women from receiving social support, accessing other reproductive health services such as postpartum care and being able to negotiate safer sex [20,21].

In 2006, the WHO issued a meeting report on addressing violence against women in HIV testing and counselling services [22]. This report called for operational research to assess counselling, communication and referral tools to support women through the disclosure and risk-reduction planning processes. The report identified multiple models for integration in this area, including in HIV testing and counselling protocols that considered risk of violence as part of efforts to evaluate the safety of HIV status disclosure. However, the report did not assess evidence of success of these programmes. To examine the evidence for such programmes nearly a decade after the release of this report, we conducted a systematic review of rigorous evaluations of interventions to facilitate safe disclosure of HIV status for women living with HIV who fear violence or are currently experiencing violence.

## Methods

We conducted a systematic review of the literature following PRISMA guidelines [23].

### Inclusion criteria

Inclusion criteria for the review were as follows:

1. Published in a peer-reviewed journal, presented as an abstract at a scientific conference or presented as a grey literature report prior to the search date of 1 April 2015
2. Comparative study (including either pre-/post- or multi-arm comparison groups) assessing one or more interventions to facilitate safe disclosure of HIV status for women living with HIV who fear violence or who disclose that they are currently experiencing violence compared with no intervention or standard of care
3. Measures one or more of the following outcomes: (1) disclosure, (2) violence (physical, sexual, emotional), (3) fear of violence, (4) other adverse events (e.g. relationship dissolution, abandonment, job loss, loss of children, loss of access to services, etc.) or (5) positive outcomes (e.g. feelings of individual empowerment, safety, partner involvement, better physical health for self and children, HIV care and treatment engagement, adherence to antiretroviral treatment, etc.)

Following Obermeyer et al. [7], we defined *HIV serostatus disclosure* as “the process of revealing a person's HIV status.” However, while Obermeyer et al.'s definition encompasses both HIV-positive and HIV-negative serostatus disclosure, we focused on disclosure outcomes among HIV-positive women where such data were available. We included studies among all populations of women living with HIV, including adolescents (10 to 19 years) and young people (20 to 24 years) [24] and women who are members of key populations (e.g. sex workers, women who use drugs, and women in prisons or other closed settings). We also included all women living with HIV, not just those who are newly diagnosed through HIV testing services.

Interventions to facilitate safer disclosure of HIV status were defined as any effort to reduce the risk of violence or other negative outcomes associated with HIV serostatus disclosure. Such efforts could include identifying people at risk of violence following disclosure, helping them find strategies to disclose in a way that reduced their risk of violence (such as through facilitated disclosure, role plays or safe disclosure plans) or helping them decide when non-disclosure was the safest option. Studies examining all types of violence were included, including physical, sexual and emotional violence, and all types of violence perpetrators, including intimate partners, family members and others. Studies could assess violence using any measure (e.g. the conflict tactic scale, WHO methodology, DHS methodology or an alternate measure). We defined current experience of violence as recent violence (in the past 12 months); however, we included articles that used any definition of current or recent experience of violence as long as it was intended to identify violence experienced after the intervention. We were particularly interested in outcome measures that captured violence related to HIV serostatus disclosure as opposed to background levels of violence, such as lifetime prevalence of violence.

No restrictions were placed based on location of the intervention. No language restrictions were used on the search; if we had identified articles published in languages other than English that met the inclusion criteria, we intended to translate them into English.

### Search strategy

We searched the following electronic databases through the cutoff date of 1 April 2015: PubMed, CINAHL (Cumulative Index to Nursing and Allied Health Literature) and EMBASE. The following terms were entered into all computer databases: (disclos*) AND (violence OR abuse OR rape OR “forced sex” OR “coerced sex”) AND (HIV OR AIDS). We also conducted secondary reference searching on all studies included in the review as well as other review articles on interventions to facilitate HIV serostatus disclosure [25] and key meeting reports [22]. We used Google Scholar to identify articles that cited the 2006 WHO meeting report “Addressing violence against women in HIV testing and counselling” [22]. Further, we contacted a small number of selected experts in the field, including the lead authors of included studies, to identify any additional studies we may have missed.

We searched for conference abstracts by searching websites for the following conferences through 1 April 2015: International AIDS Conference; IAS Conference on HIV Pathogenesis, Treatment, and Prevention; the Conference on Retroviruses and Opportunistic Infections; the International Conference on AIDS and STIs in Africa; and the Sexual Violence Research Initiative (SVRI) conference. We attempted to include abstracts from the End Violence Against Women International Annual Conference and the World Association for Sexual Health Congress, but they were not available online.

To search for other grey literature, we searched the USAID Development Experience Clearinghouse (DEC) under the terms *HIV* and *violence* and reviewed websites of the following organizations known to be involved in initiatives related to violence and HIV: SVRI, the STRIVE research consortium, London School of Hygiene and Tropical Medicine – Gender Violence and Health Centre, Futures without Violence, the Futures Group, Columbia University ICAP programme, International Planned Parenthood Federation, FHI360, MenEngage, EngenderHealth and Population Council.

### Screening abstracts

Citations identified through the search strategy underwent an initial screening by a single reviewer based on title and abstract. All citations that were considered possibly relevant were then screened by two reviewers separately to assess whether they met the inclusion criteria, with differences resolved through consensus. Full text articles were obtained of all selected abstracts and two independent reviewers made a final determination of study inclusion after full text review.

### Data extraction and analysis

Data were extracted independently by two reviewers using standardized forms. Differences in data extraction were resolved through consensus and discussion with all authors when necessary. The following information was gathered from each included study: citation information, study objectives, location, population characteristics, description of the intervention, study design, sample size, follow-up periods and loss to follow-up, analytic approach, violence measures, outcome measures, comparison groups, effect sizes, confidence intervals (CIs), significance levels, conclusions and limitations. For randomized controlled trials, risk of bias was assessed using the Cochrane Collaboration's tool for assessing risk of bias [26].

Meta-analysis was not conducted due to the small number of included studies. Instead, we present a descriptive summary of the findings across studies based on our coding categories and outcomes.

## Results

**Figure 1:**
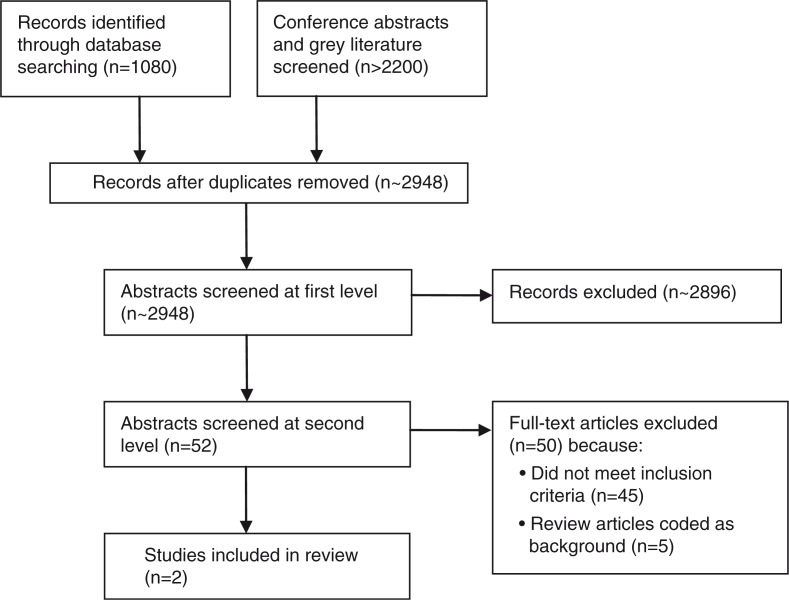
PRISMA flowchart showing disposition of citations through the search and screening process.

Our initial database search yielded 1080 published citations (Figure 1). In addition, over 2200 conference abstracts, unpublished reports and other grey literature publications were reviewed. The vast majority of these were excluded in the initial screening; 52 were retained for closer consideration by two independent reviewers. At this level, 45 citations were excluded for not meeting the inclusion criteria – generally because they did not report specific intervention evaluation data related to safer HIV disclosure – and five were literature reviews included as background material. We identified two studies that ultimately met the inclusion criteria. One of the studies published evaluation results in 2015 [27], and the other is completed [28] but evaluation results for the safer disclosure component have not yet been published. The lead authors of both studies were contacted to obtain additional information.

### Study descriptions

Table 1 presents descriptions of the included studies. Both were conducted in sub-Saharan Africa, one in rural Uganda [27] and one in urban South Africa [28]. Both integrated screening for intimate partner violence (IPV) into HIV testing and counselling and trained counsellors to facilitate discussions about disclosure based on a woman's risk of violence. In addition, both studies included multiple other intervention components designed to address community norms around IPV and HIV [27] or to enhance services for women undergoing HIV testing [28].

**Table 1:** Descriptions of evaluated interventions

Study	Setting	Population characteristics	Intervention description
Wagman et al., 2015	Uganda Rakai district	General adult population Gender: 59% female, 41% male Age range: 16 to 42 years	**Safe disclosure component:** The Safe Homes and Respect for Everyone (SHARE) project included a screening and brief intervention to reduce HIV-disclosure-related violence and sexual risk in women seeking HTC. HIV counselling protocols were modified to address IPV, and HTC counsellors were trained to screen women for IPV and handle or refer IPV cases; help HIV-positive women develop safe HIV disclosure plans; and help abused women develop safe sex negotiation skills. The intervention was designed for women newly diagnosed with HIV, but was offered to women who were diagnosed previously but had not disclosed their status and expressed fear of violence. **Other components:** SHARE also included extensive community-based mobilization to change attitudes and social norms that contribute to IPV and HIV risk.
Maman et al., 2014 and in progress	South Africa Umlazi Township, Durban	Pregnant women Gender: 100% female Mean age: 25.5 years	**Safe disclosure component:** The South Africa HIV/AIDS Antenatal Post-test Support (SAHAPS) study included an additional structured discussion tool to help women assess their risk for physical harm following disclosure in women seeking HTC. The counselling tool consisted of five questions that were meant to prompt discussion about the possibility of disclosure-related violence between the counsellors and their clients. Based on answers to these questions, counsellors were trained to explore women's risk of disclosure-related violence and potentially explore alternative options including opting not to disclose, deferring disclosure to a time when women's safety can be insured or developing a plan for mediated disclosure in which the women either brings the partner to the clinic to disclose in the presence of a counsellor or identifies a trusted family member or friend who can be present with the woman when she shares her HIV test results with her partner, or alternative options. **Other components:** SAHAPS also included enhanced HIV pre- and post-test counselling at the first antenatal visit, as well as two additional counselling sessions at 6 and 10 weeks postpartum. The postpartum counselling sessions were conducted by the same counsellors the women saw during their antenatal visit. Women also had the option to use support groups, which were ongoing through the antenatal and postpartum period, and the option to use onsite legal services.

HTC, HIV testing and counselling; IPV, intimate partner violence.

In the rural Rakai district, Uganda, the Safe Homes and Respect for Everyone (SHARE) project provided a combination of IPV prevention and HIV services from 2005 to 2009 [27]. SHARE employed two main approaches to achieve these goals: a screening and brief intervention in HIV testing and counselling to reduce HIV disclosure-related violence and sexual risk and community-based mobilization to change attitudes and social norms that contribute to IPV and HIV risk [29]. For the safer disclosure component, counsellors were trained to screen women for IPV and handle or refer IPV cases, help HIV-positive women develop safe HIV disclosure plans and help abused women develop safe sex negotiation skills. Additional community mobilization activities included advocacy with local leaders, officials and policy makers; capacity building and activism with community members and volunteers; dissemination of booklets, brochures, posters, story cards and other learning materials; special events such as fairs, marches, campaigns and poster shows; work with youth and men; and support groups for HIV-positive women. The intervention was evaluated through a cluster-randomized trial nested within the Rakai Community Cohort Study – an open, community-based cohort [27].

In Umlazi Township, Durban, South Africa, the South Africa HIV/AIDS Antenatal Post-Test Support (SAHAPS) study evaluated an enhanced counselling intervention for pregnant women undergoing HIV testing and counselling [28]. The intervention group received enhanced pre- and post-test counselling during antenatal care as well as two additional counselling sessions at 6 and 10 weeks postpartum. Women also had the option to use support groups and onsite legal services. For the safer disclosure component, counselling included a five-question structured discussion tool to help women assess their risk for physical harm following disclosure. Counsellors were then trained to explore women's risk of disclosure-related violence and to explore alternative options where indicated. The intervention was evaluated through an individual randomized controlled trial [28].

### Study results

Table 2 presents a summary of key characteristics and outcomes for the included studies. Both studies received generally high marks on the Cochrane risk of bias tool, although due to the nature of the interventions, neither study was able to blind participants or study staff to intervention allocation. Both studies examined the impact of the intervention on disclosure and violence; both measured violence using an adapted version of the conflict tactics scale. The SAHAPS study measured disclosure and violence 14 weeks after the receipt of HIV test results and excluded women who already knew they were HIV-positive, enabling measurement of violence that was proximal to disclosure. In contrast, the SHARE study measured disclosure and violence in the past year among community members residing in the intervention and control communities, including people who did not receive new HIV results during the time of the intervention. Neither study measured the effect of the intervention on fear of violence, other adverse events or positive outcomes. Neither study reported outcomes specifically among women living with HIV who were identified as experiencing or fearing violence who received the safer disclosure intervention; however, the SHARE study was able to provide unpublished data for the sub-sample of women living with HIV. Below, we present outcomes of both studies on disclosure and violence among women.

**Table 2:** Study characteristics and outcomes among women

Study	Setting	Study population	Study design	Main findings on disclosure	Main findings on violence
Wagman et al., 2015	Uganda Rakai district	Total study population: *N*=11,448 (5337 intervention, 6111 control) general adult population (59% women, 41% men) HIV-positive women: *N*=791 (343 intervention, 448 control)	Cluster randomized controlled trial *N*=4 intervention clusters; 7 control clusters. Assessments took place at baseline, 16 months and 35 months follow-up.	***Among all women:*** Reported disclosure was significantly higher among women in intervention compared to control communities at 35 months (42%, 37%; aPRR=1.15 [95% CI 1.06, 1.24]), but was not significantly different at 16 months. ***Among HIV***+***women:*** Reported disclosure was higher, but not statistically significantly different, among HIV+ women in intervention compared to control communities at 16 months (27%, 25%, *p=*0.34) or 35 months (33%, 29%; *p*=0.24); PRR=1.08 (95% CI 0.82, 1.43).	***Among all women*** **a** ***:*** Reported physical violence was significantly lower among women in intervention compared to control communities at 35 months (12%, 16%; aPRR=0.79 [95% CI 0.67, 0.92]), but was not significantly different at 16 months. Reported sexual violence and forced sex were significantly lower among women in intervention compared to control communities at 35 months (10%, 13%; aPRR=0.80 [CI: 0.67, 0.97]; 8%, 11%; aPRR=0.79 [CI: 0.65, 0.96]), but were not significantly different at 16 months. No significant difference in reported emotional violence at 16 or 35 months.
Maman et al., 2014, and in progress	South Africa Umlazi Township, Durban	Total study population *N*=1480 (733 intervention, 747 control) pregnant women receiving antenatal care HIV-positive women: *N*=571	Individual randomized controlled trial. Assessments took place at baseline (first antenatal care visit), 14 weeks postpartum and 9 months postpartum.	***Among all women*** **a** ***:*** No significant differences in reported disclosure rates among women between intervention and control groups at 14 weeks postpartum.	***Among all women*** **a** ***:*** No significant differences in reported physical, emotional or sexual violence among women between intervention and control groups at 14 weeks postpartum.

PRR: prevalence rate ratio; aPRR: adjusted prevalence rate ratio; CI: confidence interval (all are 95% confidence intervals).

a Data were not available among HIV-positive women.

In the SHARE study, women in the intervention group reported higher rates of HIV serostatus disclosure than women in the control group at the second follow-up; the adjusted prevalence rate ratio (aPRR) was 1.15 (95% CI: 1.06 to 1.24). Women's reports of past-year physical IPV, sexual IPV and forced sex were lower in the intervention group compared to the control group at the second follow-up (35 months after baseline) (physical IPV: aPRR: 0.79, 95% CI: 0.67 to 0.92; sexual IPV: aPRR: 0.80, 95% CI: 0.67 to 0.97; forced sex: aPRR: 0.80, 95% CI: 0.65 to 0.96). Reports of emotional IPV were not statistically significantly different at second follow-up, nor were any outcomes significant at the first follow-up (16 months after baseline).

The SHARE study authors provided additional data for women living with HIV in the sample. These data reflect women with prevalent infection, rather than just those who were newly learning their HIV-positive status (the main target of the safer disclosure intervention), and they reflect all women from the sample in the intervention communities rather than just women who received the safer disclosure intervention. At baseline, there was no difference in disclosure among women living with HIV across study arms. At both follow-ups, a higher proportion of women living with HIV in the intervention group reported disclosing compared to the control group, but this difference was not significant (prevalence rate ratio for entire follow-up period: 1.08; 95% CI: 0.82 to 1.43).

While the SAHAPS study has not yet published disclosure and violence outcomes, the study authors provided additional data showing that there were no significant differences across study arms in either disclosure or IPV at the 14 week postpartum assessment. While the effect of the safer disclosure component cannot be isolated from the effect of the rest of the intervention, the fact that the study saw no measurable effect on disclosure or violence overall may also suggest that the individual components had no measurable effect. The authors also looked at interactions between HIV status, disclosure and IPV among participants in both study arms at follow-up. Among women who disclosed, IPV at 14 weeks postpartum was not significantly different for HIV-positive and HIV-negative women. However, among women who had not disclosed, the odds of reporting IPV at 14 weeks was almost five times higher for HIV-positive women as compared to HIV-negative women.

## Discussion

Despite significant attention to the intersections between HIV and violence as well as international policy consensus on the need to facilitate safer disclosure of HIV serostatus for women who experience or fear violence, we identified only two studies evaluating such interventions globally. Both studies were from sub-Saharan Africa and employed strong randomized designs. However, neither provided clear evidence for the effectiveness of a safer disclosure intervention as they were not designed to isolate the contribution of the safer disclosure components from the broader multiple-component interventions. The evidence base for interventions to facilitate safer disclosure is thus quite limited, and further studies are needed.

For all women, the SHARE study in Uganda reported positive outcomes for disclosure and violence at the second follow-up. Unpublished data among HIV-positive women showed non-significant but positive trends in disclosure. However, these analyses could not distinguish women who had received the enhanced counselling intervention from those who had not or newly diagnosed women from those who had known their HIV-positive status for a long time, both of which would likely attenuate an effect toward the null. In the SAHAPS study in South Africa, unpublished data showed that women who were exposed to the enhanced counselling intervention were no more likely to disclose to their partner or report violence after HIV diagnosis than women who received standard of care HIV testing services. Additional analyses suggested that women who were already at higher risk of IPV – particularly HIV-positive women – chose not to disclose despite, or perhaps because of, the safer disclosure intervention, while women who disclosed were those who already knew it was safe to do so. This interpretation is supported by previous descriptive studies showing that women who have disclosed their status are less likely to have ever experienced violence [30] and a systematic review of male involvement in PMTCT programmes, which found that women who want to test together as couples may be a self-selected group of those already in non-violent relationships [31]. Together, these findings suggest that while IPV continues to be a significant barrier to disclosure for women who fear violence, it has not been adequately addressed in current approaches related to provision of HIV testing, treatment and care services in healthcare settings. There are several options to be considered in such a scenario. One option is that in the absence of specific interventions to respond to violence or promote safety, women who are at risk of violence may be better off being supported in a decision not to disclose their status. Another consideration is to promote safety for women who do want to disclose or who may experience inadvertent disclosure of their status and to do so in line with WHO guidelines. These guidelines recommend training of healthcare providers, especially in HIV testing settings, to identify women who are at risk of IPV and offer a response that includes first-line psychological support including safety planning, addressing immediate needs for physical and mental health and providing referrals to appropriate services that address violence [32].

Both studies included in this review evaluated interventions that integrated screening for IPV into HIV testing services and trained counsellors to facilitate discussions about disclosure based on a woman's risk of violence. HIV testing services provide an opportunity for safer disclosure interventions, but this is not the only potential approach to facilitating safer disclosure for women who experience violence. The 2006 WHO consultation found that programmes jointly addressing the problems of HIV and violence used two broad types of strategies: those addressing violence against women in HIV testing programmes and those addressing HIV-related needs among women who experience violence [22]. While both of the interventions identified in this review integrated violence assessments with HIV services, there may be other interventions addressing violence risk in the context of HIV serostatus disclosure outside of testing that have not been evaluated. Creative ideas include integrating safer disclosure messages into support groups for people living with HIV and training peer counsellors to address disclosure-related violence as part of their work. We encourage programmes currently attempting to intervene to facilitate safer disclosure of HIV status for women who experience or fear violence to share their results in implementation science journals, short report formats or by other means of dissemination to add to the existing evidence base.

In this review, we included all studies that attempted to directly address safer HIV serostatus disclosure for women who experience or fear violence. We did not include studies that may indirectly encourage safer disclosure. For example, many violence prevention programmes that seek to change community norms around violence give women legal rights and protections and give women greater control over financial resources may also have the effect of creating safer environments for women to disclose their serostatus. Similarly, interventions that attempt to reduce HIV-related stigma and discrimination may also reduce the risk of violence for women living with HIV who choose to disclose. Efforts to change community norms that justify or tolerate violence against women and that perpetrate HIV-related stigma are critical to creating enabling environments that reduce violence, which in turn can contribute to facilitating safer disclosure [33]. However, our review focused on direct interventions to facilitate safe HIV disclosure that can be implemented within the context of existing HIV services.

Our findings must be seen in the context of the limitations of our review. While we did not include any language restrictions in our inclusion criteria and searched several online databases that include articles in languages other than English, our search for grey literature only included English-language websites as we were not familiar with any relevant websites in other languages. We may therefore have missed some unpublished evaluations of interventions in other languages.

While HIV serostatus disclosure may lead to many positive outcomes, for many women living with HIV, fear of violence in response to HIV serostatus disclosure is a serious concern. In many settings, health workers are not trained to identify women at risk of violence, provide them with appropriate care and support them in enhancing their safety in relation to health behaviours (e.g. disclosure, safer sex). Moreover, in many settings there are few support services or referral options for women who experience violence generally, regardless of their HIV status. This situation poses a challenge for all health services, including HIV and reproductive health programmes, which are often resource-constrained at the human and financial levels and may not be able to provide comprehensive violence services. Further, as new approaches to HIV testing are expanded as part of efforts to reach the UNAIDS 90-90-90 targets, including community-based and provider-initiated testing, self-testing and testing conducted by lay healthcare providers [34], programmes will need to consider how to achieve safer disclosure of HIV status within the complexities inherent in each of these models [35]. Further research is needed to identify which interventions can best achieve the objective of supporting women living with HIV who experience or fear violence to safely disclose their HIV serostatus – or not to disclose at all, as appropriate – in order to inform programme and policy decisions.
